# Supplementary material for: Assessing COVID-19 Vaccine Booster Hesitancy Using the Modified 5C Scale in Zhejiang Province, China: A Cross-Sectional Study
Source: Vaccines (Basel). 2023 Mar 21;11(3):706. doi: 10.3390/vaccines11030706 (PMC10056561; doi:10.3390/vaccines11030706)
Supplement: Supplementary file 1 [file vaccines-11-00706-s001.zip › vaccines-2227033-supplementary.pdf]

Supplementary Table S1. The modified 5C scale

Strongly agree=1, agree=2, not sure=3, disagree=4 or strongly disagree=5

COVID-19 Booster Vaccine Hesitancy Scale Items	1	2	3	4	5
**Dimension 1: Confidence**					
1. COVID-19 booster vaccines are safe.					
2. COVID-19 booster vaccines are effective.					
3. Regarding the booster campaign initiated by government, I am confident that public authorities decide in the best interest of the community.					
**Dimension 2: Complacency**					
4. The booster is unnecessary because the government takes strict prevention and control measures against COVID-19 which is not prevalent in China so far.					
5. My immune system is so strong, it can protect me against COVID-19.					
6. The COVID-19 is not so severe that I should get the booster.					
**Dimension 3: Constraint**					
7. Everyday stress prevents me from getting the booster.					
8. For me, it is inconvenient to receive the booster.					
9. Primary vaccination experience was not good which makes me feel unwilling to get the booster.					
**Dimension 4: Calculation**					
10. When I think about getting the booster, I weigh benefits and risks to make the best decision possible.					
11. For each vaccination, I closely consider whether it is useful for me.					
12. It is important for me to fully understand relative messages about the booster vaccination before I get vaccinated.					
**Dimension 5: Collective Responsibility**					
13. I get vaccinated because I can protect people with a weaker immune system.					
14. Vaccination is a collective action to prevent the spread of COVID-19.					

Supplementary Table S2. The complete 30-item questionnaire for formal investigation

Section I: General Information.

15. The gender A. Male B. Female C. Prefer not to say
16. How old are you (Years old)?<br>A. <18 B. 18-30 C. 31-40 D. 41-50 E. 51-60 F. >=60
17. What's your residence? ___________City_____________District/County
18. Your residence belong to? A. Urban B. Rural
19. What is your highest degree of education level (including current enrollment)?<br>A. Middle school and below B. High school C. Bachelor D. Master and above
20. What is your marital status?

A. Married B. Unmarried C. Divorced D. Death of a spouse
21. What is your annual income (Yuan/RMB)?<br>A. Below ¥50k B. ¥50k-100k C. ¥100k-150k D. ¥150k-200k E. ¥200k and above
22. What is your occupation type?<br>A. Civil servant and technical personnel<br>B. Medical worker<br>C. Manufacturing and commercial worker<br>D. Public service worker<br>E. Farmer/Herder/Fisherman<br>F. Unemployed/Retiree<br>G. College student<br>H. Others_______

Section II: Information on primary doses against COVID-19.

Please check through "Zheliban" app developed by the General Office of the People's Government of Zhejiang Province or "Alipay" app developed by Alipay Technology.

23. Have you completed the primary doses against COVID-19 yet?<br>A. Yes B. No C. Not sure.
24. What is your vaccine type of your primary doses?<br>B. Inactivated (Sinovac)<br>C. Inactivated (Sinopharm)<br>D. Viral vector<br>E. Protein subunit<br>F. mRNA<br>G. Unclear

Section III: Booster hesitancy with the modified 5C scale.

Strongly agree=1, agree=2, not sure=3, disagree=4 or strongly disagree=5

**COVID-19 Booster Vaccine Hesitancy Scale Items**	**1**	**2**	**3**	**4**	**5**
25. COVID-19 booster vaccines are safe.					
26. COVID-19 booster vaccines are effective.					
27. Regarding the booster campaign initiated by government, I am confident that public authorities decide in the best interest of the community.					
28. The booster is unnecessary because the government takes strict prevention and control measures against COVID-19 which is not prevalent in China so far.					
29. My immune system is so strong, it can protect me against COVID-19.					
30. The COVID-19 is not so severe that I should get the booster.					
31. Everyday stress prevents me from getting the booster.					
32. For me, it is inconvenient to receive the booster.					
33. Primary vaccination experience was not good which makes me feel unwilling to get the booster.					
34. When I think about getting the booster, I weigh benefits and risks to make the best decision possible.					

COVID-19 Booster Vaccine Hesitancy Scale Items	1	2	3	4	5
35. For each vaccination, I closely consider whether it is useful for me.					
36. It is important for me to fully understand relative messages about the booster vaccination before I get vaccinated.					
37. I get vaccinated because I can protect people with a weaker immune system.					
38. Vaccination is a collective action to prevent the spread of COVID-19.					

Section IV: Booster willingness.

39. Are you willing to receive the booster dose of COVID-19 vaccines?

A. Definitely go to get vaccinated.
B. Likely to get vaccinated.
C. Not sure
D. Unlikely to get vaccinated.
E. Definitely not to get vaccinated.

40. What are your reasons if you would like to get the booster (**Multiple choice, only for those choose A, B in Question 24**)?

A. So far, two-dose series of primary doses might not provide sufficient protection.
B. I am immunocompromised or have underlying diseases who need the booster to enhance protection.
C. The booster is safe which I want to get to enhance protection.
D. Get the booster can make me feel safer.
E. Get the booster make my daily activities (work and life) more convenient.
F. Get the booster can also protect others, which contributes to herd immunity.
G. Booster is required/encouraged by community or government.
H. Good previous vaccination experience for primary doses against COVID-19.
I. Impact of people around or recommended by professionals (medical staff, medical experts, etc).
J. Other reasons____

41. What are your reasons if you would **not** like to get the booster (**Multiple choice, only for those choose C, D, and E in Question 24**)?

A. The risk of infection is low in China right now.
B. The disease severity caused by COVID-19 is largely decreasing.
C. Two-dose series in primary vaccination is sufficient to provide protection.
D. There is no need for me to get vaccinated if other people have gotten the booster.
E. Safety concern about the booster.
F. Underlying physical conditions which are not suitable for the booster.
G. Not good experience on primary vaccination.
H. The rate of adverse effect is uncertain.
I. Inadequate health promoting materials on the booster for me.
J. Not encouraged or recommended by people around me.
K. It’s troublesome and inconvenient for me the get the booster.
L. Too busy to get the booster.
M. Other reasons____

Section V: Previous experience on primary doses.

42. How do you feel about your vaccination experience of primary doses against COVID-19?<br>A. Very satisfied<br>B. Satisfied<br>C. Not sure<br>D. Disappoint<br>E. Very disappoint
43. What are your reasons if your experience is good (**Multiple choice, only for those choose A, B and C in Question 27**)?<br>A. Good medical skills during vaccination.<br>B. The environment of vaccination site is quiet and hygienic, which makes me feel safe.<br>C. Convenient and high accessibility to get vaccinated.<br>D. The process of getting vaccinated is clear and reasonable, and the queue is orderly.<br>E. No adverse effect after the primary doses.<br>F. My daily activities are convenient after vaccination.<br>G. Other reasons____
44. What are your reasons if your experience is not good (**Multiple choice, only for those choose D and E in Question 27**)?<br>A. Not good medical skills during vaccination.<br>B. Not good environment of vaccination site, which is crowed/noisy/unhygienic/sweltering, and so on.<br>C. I have adverse reaction after injection.<br>D. Strong recommendation for vaccination by communities or government is troublesome.<br>E. Unintelligent online appointment of vaccination.<br>F. Long waits.<br>G. Vaccine shortage.<br>H. Inconvenient to get vaccinated due to daily restrictions.<br>I. Other reasons____

Supplementary Table S3. Pairwise comparisons based on *Bonferroni correction* method between booster hesitancy and various significant factors in univariate analysis.

Item	Pairwise comparison groups		Pairwise hesitancy rate (%) for each group		*P.adj**
Demographic characteristics					
**Age (Years)**					
	31-40	61-	133 (12.41)	88 (18.22)	0.028
**Educational status**					
	Middle school and below	Bachelor	136 (18.63)	338 (13.57)	0.005
**Annual income (Yuan/RMB)**					

＜50,000	100,001-150,000	289 (17.74)	89 (10.93)	<0.001
＜50,000	150,001-200,000	289 (17.74)	32 (11.03)	0.047
**Marital status**				
Married	Single	316 (13.41)	259 (16.71)	0.005
Married	Widowed	316 (13.41)	40 (76.92)	<0.001
Single	Widowed	259 (16.71)	40 (76.92)	<0.001
Divorced	Widowed	13 (16.25)	40 (76.92)	<0.001
**Occupation**				
Civil servant and technical personnel	Unemployed/Retiree	149 (11.93)	54 (19.35)	0.047
Civil servant and technical personnel	College student	149 (11.93)	166 (19.67)	<0.001
Medical worker	Manufacturing and commercial worker	51 (9.04)	90 (16.61)	0.006
Medical worker	Unemployed/Retiree	51 (9.04)	54 (19.35)	<0.001
Medical worker	College student	51 (9.04)	166 (19.67)	<0.001
Vaccine Type				
**Brand of primary Covid-19 doses**				
Inactivated (Sinovac)	Inactivated (Sinopharm)	403 (13.75)	159 (18.32)	0.006
Previous vaccination experience level of primary Covid-19 doses				
Very satisfied	Satisfied	63 (3.60)	254 (14.40)	<0.001
Very satisfied	Not sure	63 (3.60)	238 (52.89)	<0.001
Very satisfied	Disappoint	63 (3.60)	32 (55.17)	<0.001
Very satisfied	Very disappoint	63 (3.60)	11 (64.71)	<0.001
Satisfied	Not sure	254 (14.40)	238 (52.89)	<0.001
Satisfied	Disappoint	254 (14.40)	32 (55.17)	<0.001
Satisfied	Very disappoint	254 (14.40)	11 (64.71)	<0.001
5C scale dimensions				
**Confidence**				
High	Middle	471 (12.15)	116 (80.00)	<0.001
High	Low	471 (12.15)	11 (68.75)	<0.001
**Complacency**				
High	Middle	135 (25.91)	170 (43.26)	<0.001
High	Low	135 (25.91)	293 (9.38)	<0.001

	Middle	Low	170 (43.26)	293 (9.38)	<0.001
**Constraint**					
	High	Low	142 (39.78)	280 (8.66)	<0.001
	Middle	Low	176 (39.20)	280 (8.66)	<0.001
**Calculation**					
	High	Middle	441 (14.18)	134 (28.03)	<0.001
	High	Low	441 (14.18)	23 (5.10)	<0.001
	Middle	Low	134 (28.03)	23 (5.10)	<0.001
**Collective responsibility**					
	High	Middle	352 (10.46)	216 (38.50)	<0.001
	High	Low	352 (10.46)	30 (26.79)	<0.001
	Middle	Low	216 (38.50)	30 (26.79)	0.018

Note: * *P* value was adjusted based on the *Bonferroni correction* method.
